# Cardiorespiratory Coordination in Repeated Maximal Exercise

**DOI:** 10.3389/fphys.2017.00387

**Published:** 2017-06-07

**Authors:** Sergi Garcia-Retortillo, Casimiro Javierre, Robert Hristovski, Josep L. Ventura, Natàlia Balagué

**Affiliations:** ^1^Complex Systems in Sport, Institut Nacional d'Educació Física de Catalunya (INEFC), Universitat de Barcelona (UB)Barcelona, Spain; ^2^Complex Systems in Sport, School of Health and Sport Sciences (EUSES), Universitat de GironaGirona, Spain; ^3^Department Physiological Sciences, Universitat de Barcelona (UB)Barcelona, Spain; ^4^Complex Systems in Sport, Faculty of Physical Education, Sport and Health, Saints Cyril and Methodius University of SkopjeSkopje, Macedonia

**Keywords:** cardiorespiratory exercise testing, principal components analysis, coordinative variables, information entropy, exercise-induced fatigue

## Abstract

Increases in cardiorespiratory coordination (CRC) after training with no differences in performance and physiological variables have recently been reported using a principal component analysis approach. However, no research has yet evaluated the short-term effects of exercise on CRC. The aim of this study was to delineate the behavior of CRC under different physiological initial conditions produced by repeated maximal exercises. Fifteen participants performed 2 consecutive graded and maximal cycling tests. Test 1 was performed without any previous exercise, and Test 2 6 min after Test 1. Both tests started at 0 W and the workload was increased by 25 W/min in males and 20 W/min in females, until they were not able to maintain the prescribed cycling frequency of 70 rpm for more than 5 consecutive seconds. A principal component (PC) analysis of selected cardiovascular and cardiorespiratory variables (expired fraction of O_2_, expired fraction of CO_2_, ventilation, systolic blood pressure, diastolic blood pressure, and heart rate) was performed to evaluate the CRC defined by the number of PCs in both tests. In order to quantify the degree of coordination, the information entropy was calculated and the eigenvalues of the first PC (PC1) were compared between tests. Although no significant differences were found between the tests with respect to the performed maximal workload (Wmax), maximal oxygen consumption (VO_2_ max), or ventilatory threshold (VT), an increase in the number of PCs and/or a decrease of eigenvalues of PC_1_ (*t* = 2.95; *p* = 0.01; *d* = 1.08) was found in Test 2 compared to Test 1. Moreover, entropy was significantly higher (*Z* = 2.33; *p* = 0.02; *d* = 1.43) in the last test. In conclusion, despite the fact that no significant differences were observed in the conventionally explored maximal performance and physiological variables (Wmax, VO_2_ max, and VT) between tests, a reduction of CRC was observed in Test 2. These results emphasize the interest of CRC evaluation in the assessment and interpretation of cardiorespiratory exercise testing.

## Introduction

The effects of graded exercise testing on isolated physiological variables (e.g., ventilation, VE; oxygen consumption, VO_2_; blood pressure, BP, and heart rate, HR) have been widely described in the literature (see Skinner and McLellan, 1980 for a review). The quantification of the maximal and threshold values of these variables together with the maximal performed workload is a common goal of cardiorespiratory testing and the functional assessment of all types of populations (American Thoracic Society, 2003). Although it is well-known that metabolic, cardiovascular, and pulmonary functions work in coordination, few attempts have been made to study cardio-respiratory coordination during exercise, and specifically as a consequence of repeated maximal exercise bouts.

As physiological systems are interdependent and display nonlinear dynamics, they are better approached through time series and complex systems methodologies (Schulz et al., 2013). To study the coordinated behavior of a system with multiple degrees of freedom, complex dynamic approaches propose the detection of the so-called coordinative or collective variables. These variables are characterized as capturing the coordinated behavior of the system under study (Haken, 2000) without dividing it into separated components. One strategy to reduce the dimensionality of a system with multiple degrees of freedom and detect coordinative variables is to apply a principal component analysis (PCA). A PCA approach has been proposed to reduce the dimensionality of the physiological response to exercise identifying the variables that co-vary in time during cardiorespiratory testing (Balagué et al., 2016). Variables that co-vary are those that have correlated increments and decrements, i.e., share their variance forming a single dimension, a coordinative variable or a principal component (PC). In this way the cardio-respiratory response can be represented using few principal components (PCs). PCs are extracted in decreasing order of importance so that the first PC (PC_1_) accounts for as much of the variation as possible, with each successive PC (PC_2_, PC_3_, etc.) accounting for a little less (Balagué et al., 2016). The number of PCs reflects the dimensionality or degree of cardiorespiratory coordination (CRC), so that a decrease is indicative of greater coordination (a higher degree of co-varying physiological variables in time), and an increase of lower coordination (a lower degree of co-varying physiological variables in time). Balagué et al. (2016) found a reduction in the number of PCs during cardiorespiratory testing after training. However, these coordinative changes were not reflected in the commonly registered physiological and performance variables such us maximal workload (Wmax), maximal oxygen consumption (VO_2_ max), or ventilatory threshold (VT), which could be less sensitive to training effects, as indicated by the authors.

Together with the PCA approach, the information entropy measurement (*information compression*; Haken, 2000) is used by complex systems methodologies to quantify the number of coordinative states that are accessible to a system (e.g., the human body; Seely and Macklem, 2012) and the minimum information needed to identify its current state (Naudts, 2005). Entropy increases when more states are available, i.e., when more information is needed to specify the system's state. The larger number of available states is a direct consequence of the decreased co-variation among the cardiorespiratory variables, changing more independently from one another. It has been recently used to analyse CRC in several research fields, such as obstructive sleep apnea (Chang et al., 2013), online gaming (Chang et al., 2015), mental stress (Widjaja et al., 2015), or to investigate thought dynamics during an exhausting cycling exercise (Balagué et al., 2014). Nevertheless, entropy has not been used together with the PCA approach to analyse CRC within exercise settings.

Repeated bouts of maximal exercise increase the level of fatigue, but do not seem to affect the reliability of maximal physiological variables (like VO_2_ max) and lack of adverse effects in healthy subjects (Hall-López et al., 2015). Thus, consecutive cardiorespiratory exercise testing seems a convenient procedure to test the sensitivity of CRC. The reciprocal compensation of systems functions to attain the same performance, called synergy, is a well-known coordination property of biological systems (Scholz and Schöner, 1999; Latash, 2008). Applied to physiological systems it means that they can be organized in differently structured coalitions in order to re-allocate resources and maintain performance variables constant (e.g., when blood pressure falls to a certain degree HR increases). Although motor coordination changes have been reported as a consequence of fatigue produced by repeated bouts of exercise (Mizrahi et al., 2000; Hristovski and Balagué, 2010; Vázquez et al., 2016; Bruce et al., 2017), there is no evidence regarding exercise-induced fatigue effects on CRC. Thus, the aim of this exploratory study was to delineate the behavior of CRC under different physiological initial conditions produced by two consecutive maximal exercises, evaluated in healthy adults during cardiorespiratory exercise testing. We specifically hypothesized an increase in the number of PCs (i.e., a reduction of CRC), and/or an increase in the information entropy measure (i.e., a lower degree of covariation within the system) in the second test (Test 2) compared to the first test (Test 1), without significant changes in maximal performance and maximal cardiorespiratory variables.

## Materials and methods

### Participants

Fifteen healthy adults (six males and nine females; age 22.5 ± 3.1 years, height 175.7 ± 6.9 cm, mean body mass 72.3 ± 6.3 kg and mean body mass index 23.4 ± 1.9 kg· m^−2^), with no sport specialization but who engaged in a wide range of aerobic activities at least three times a week, volunteered to participate in the study. Exclusion criteria consisted of any condition that could prevent the performance of a maximal exercise protocol. The experiment was approved by the Clinical Research Ethics Committee of the Sports Administration of Catalonia, and carried out according to the Helsinki Declaration. Participants read the study's description and risks and signed an informed consent before taking part in the study.

### Intervention and procedure

Participants performed in the same session and underwent, consecutively, two graded, and maximal cycling tests (see below). Test 1 was performed without any previous exercise and Test 2 6 min after Test 1 in order to repeat the maximal test with altered initial physiological conditions. During the 6 min recovery period the participants remained sitting on the cycle ergometer.

The incremental cycling test (Excalibur, Lode, Groningen, Netherlands) was performed using the following procedure:

The test started at 0 W and the workload was increased by 25 W/min in males and 20 W/min in females until they were not able to maintain the prescribed cycling frequency of 70 rpm for more than 5 consecutive seconds. During the test, participants breathed through a valve (Hans Rudolph, 2700, Kansas City, MO, USA) and respiratory gas exchange was determined using an automated open-circuit system (Metasys, Brainware, La Valette, France). Oxygen and CO_2_ content and air flow rate were recorded breath by breath. Before each trial, the system was calibrated with a mixture of O_2_ and CO_2_ of known composition (O_2_ 15%, CO_2_ 5%, N2 balanced; Carburos Metálicos, Barcelona, Spain), as well as with ambient air.

Haemodynamic information was obtained from participants using non-invasive finger cuff technology (Nexfin, BMEYE Amsterdam, Netherlands). The Nexfin device provides continuous blood pressure (BP) monitoring from the resulting pulse pressure waveform, and calculates both systolic and diastolic blood pressure (SBP and DBP). Participants were connected by wrapping an inflatable cuff around the middle phalanx of the finger. The finger artery pulsing is “fixed” to a constant volume by application of an equivalent change in pressure against the blood pressure, resulting in a waveform of the pressure (clamp volume method). The measurement was performed in the non-dominant arm; relaxed, supported by a measurement cable attached by a rubber band. We enabled continuous monitoring finger photoplethysmography, which is useful for assessing acute BP changes (Eckert and Hornskotte, 2002). Electrocardiogram (ECG) was continuously monitored (DMS Systems, DMS-BTT wireless Bluetooth ECG transmitter, and receiver, software DMS Version 4.0, Beijing, China). All tests were performed in a well-ventilated lab; the room temperature was 23°C and the relative humidity 48%, with variations of no more than 1°C in temperature and 10% in relative humidity. The tests were carried out at least 3 h after a light meal, and participants were instructed not to perform any vigorous physical activity for 72 h before testing (Balagué et al., 2016).

### Data analysis

To determine the sample size for this study a power analysis was conducted using G*Power 3.1 (Faul et al., 2007). Similar studies of CRC during exercise (Balagué et al., 2016) have reported medium and large effect sizes. Thus, using an effect size of *d* = 0.7, α < 0.05, power (1—β) = 0.80, we estimated a sample size = 15.

The following initial and maximal values of cardiorespiratory, performance and subjective variables were registered during both tests: initial and maximal rate of perceived exertion (RPE), initial and maximal expiratory ventilation per minute (VE), initial and maximal oxygen uptake relative to body weight (VO_2_), ventilatory threshold (VT) in % of VO_2_ max (by means of the O_2_ and CO_2_ ventilatory equivalents method; Reinhard et al., 1979), initial and maximal heart rate (HR), and maximal cycling workload (Wmax). With this purpose cardiorespiratory and performance variables were averaged every 10 s and subjective variables were registered every min, i.e., at the end of each workload. The initial values were obtained after a period of 30 min rest in the laboratory. A *t*-test or a Wilcoxon matched pairs test were used to compare initial and maximal values between Test 1 and Test 2.

In order to study CRC in each participant, the following procedure was used:

A PCA was performed on the data series of the following selected cardiorespiratory variables, in both tests: expired fraction of O_2_ (FeO_2_), expired fraction of CO_2_ (FeCO_2_), ventilation (VE), systolic blood pressure (SBP), diastolic blood pressure (DBP), and heart rate (HR). Other commonly registered variables in cardiorespiratory testing, such as respiratory equivalents, respiratory exchange ratio, oxygen pulse, oxygen consumption, etc., were excluded from the analysis due to their known deterministic mathematical relation with the aforementioned variables (Balagué et al., 2016).

In order to analyze the suitability of the implementation of the PCA, Bartlett's sphericity test and the KMO (Kaiser-Mayer-Olkin; Denis, 2016) index were calculated in each participant. The number of PCs was determined by the Kaiser-Gutmann criterion, which considers PCs with eigenvalues λ ≥ 1.00 as a significant (Jolliffe, 2002). Since the first PC (PC_1_) always contains the largest proportion of the data variance, eigenvalues and the loadings of selected cardiorespiratory variables of PC_1_ were compared between tests by means of a *t*-test.

Finally, to quantify the degree of coordination among the involved cardiorespiratory subsystems, the information entropy measure was also calculated in both tests as follows: *H* = Sum [1/2 ln(EV) + 1/2 ln (3.14) + 1/2], where *H* is the entropy of the system and EV is the PC eigenvalue (Haken, 2000). This sum encompasses all PC eigenvalues of each participant (e.g., for a participant with 2 PCs, the sum is repeated twice using eigenvalues of PC_1_ and PC_2_, respectively). A Wilcoxon matched pairs test was used to compare information entropy measure between Test 1 and Test 2. We used an alpha level of 0.05 for all statistical tests and computed effect sizes (Cohen's d) to demonstrate the magnitude of standardized mean differences.

## Results

Statistically significant differences were found regarding initial values of VE (*Z* = 3.41; *p* = 0.001; *d* = 1.32), VO_2_ (*Z* = 2.73; *p* = 0.006; *d* = 0.87), HR (*t* = 2.85 *p* = 0.01; *d* = 0.94), and RPE (*Z* = 2.39; *p* = 0.02; *d* = 0.80) between Test 1 and Test 2. However, no significant differences were found in Wmax (*t* = 1.96; *p* = 0.08), VT (*t* = 1.04; *p* = 0.32), and in maximal values of VE (*Z* = 0.04; *p* = 0.97), HR (*Z* = 0.25; *p* = 0.81), and RPE (*Z* = 1.00; *p* = 0.32; see Table 1).

**Table 1:** Means (standard deviations) of initial and maximal values of subjective estimation, cardiorespiratory variables, ventilatory threshold, and maximal workload.

	**Test 1**						**Test 2**					
	**VE (l.min-1)**	**VO_2_ (ml.kg.min-1)**	**HR (b.min-1)**	**VT (%VO_2_max)**	**Watts (W)**	**RPE**	**VE (l.min-1)**	**VO_2_ (ml.kg.min-1)**	**HR (b.min-1)**	**VT (%VO_2_ max)**	**Watts (W)**	**RPE**
Initial	10.95^*^	5.83^*^	86.20^*^	-	0.00	6.13^*^	16.29^*^	7.67^*^	102.40^*^	-	0.00	7.27^*^
	(3.16)	(1.78)	(17.28)	-	(0.00)	(0.52)	(4.65)	(2.39)	(17.37)	-	(0.00)	(1.94)
Maximal	91.78	42.44	178.86	74.70	300.67	19.60	84.94	41.65	177.85	79.49	289.67	19.62
	(35.10)	(7.14)	(5.75)	(9.70)	(85.54)	(1.06)	(26.12)	(7.68)	(8.45)	(9.64)	(84.33)	(1.12)

* *p < 0.05; VE, ventilation; VO_2_, oxygen uptake relative to body weight; VT, ventilatory threshold; HR, heart rate; RPE, rate of perceived exertion*.

**Figure 1:**
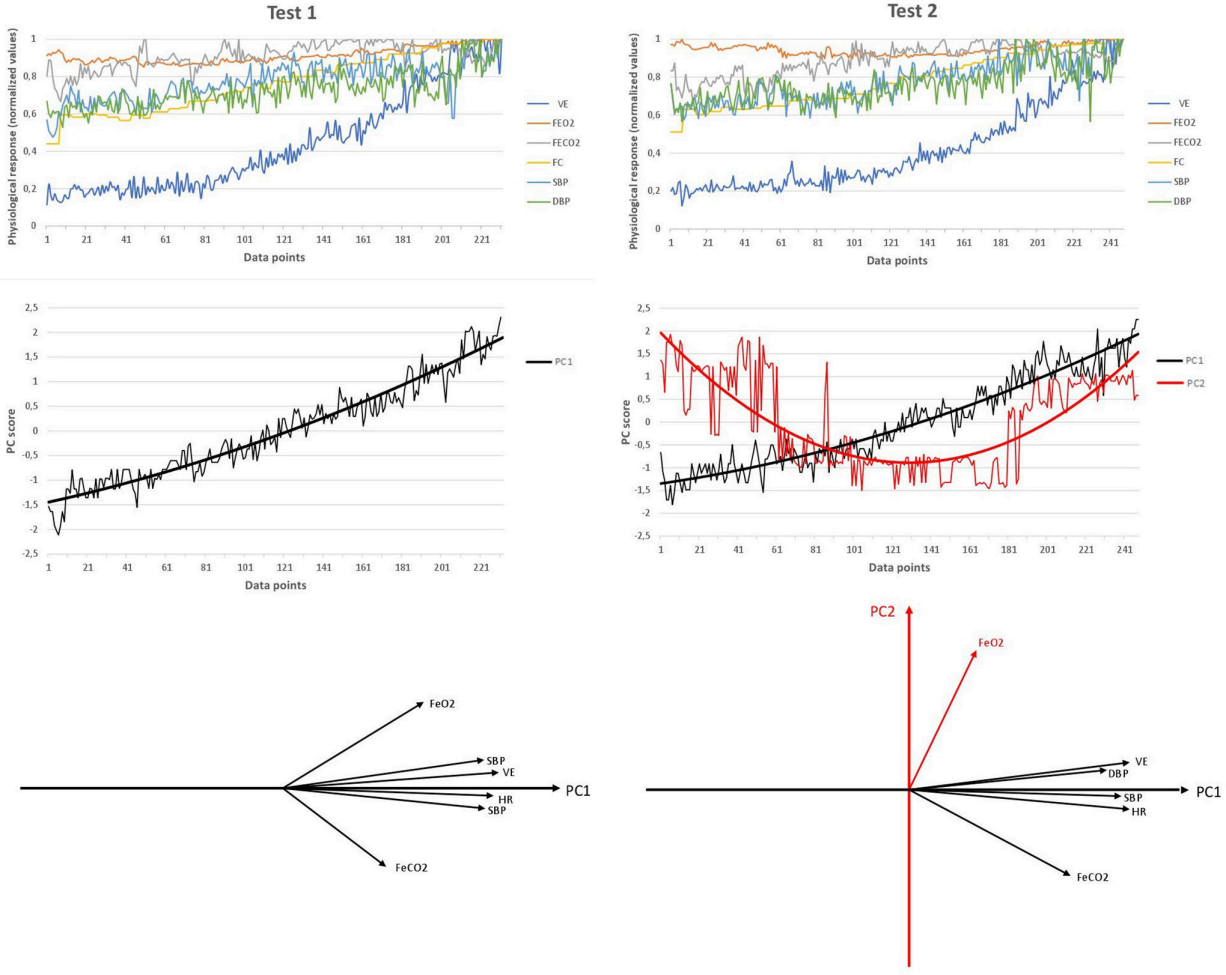
Typical example of the reduction of cardiorespiratory variables to time series of cardiorespiratory coordination variables (PCs) in Test 1 and Test 2. Top graphs: original time series of the six selected cardiorespiratory variables in Test 1 and Test 2. Middle graphs: time series of PC scores (standardized z-values in the space spanned by PCs) in both tests. The six time series are collapsed to one time series (Test 1) or two time series (Test 2) as a consequence of the PC dimension reduction. The black and the red line show the average trend of both processes as calculated by weighted least squares method. Data points of the x-axis of both graphs refers to the number of measurements recorded along the cardiorespiratory test. Bottom graphs: position of the six original cardiorespiratory vectors within the coordinate system of PCs.

A typical difference between the results of Test 1 and Test 2 is shown in Figure 1. In Test 1, the variance of the six cardio-respiratory variables was captured by a sole PC_1_, whereas in Test 2, five variables (VE, FCO_2_, HR, SBP, and DBP) showed a larger degree of co-linearity with PC_1_ and only FeO_2_ was dominantly aligned with PC_2_. Thus, PC_1_ represented CRC, and PC_2_, the idiosyncratic behavior of FeO_2_. Note that it was the shift of FeO_2_ from the PC_1_ cluster of variables that enabled the formation of PC_2_.

Bartlett's sphericity test (*p* < 0.001) and the KMO index (*M* = 0.75; *SD* = 0.05) showed a good sampling adequacy. An increase from one PC in Test 1 to two PCs in Test 2 was found in nine participants. The other six remained with the same number of PCs in Post-Et (i.e., two PCs in both tests; see Table 2). Five variables (VE, FCO_2_, HR, SBP, DBP) were always involved in forming PC_1_, whereas PC_2_ was formed by a single variable: FeO_2_. Table 3 shows the mean and *SD* of the loading of the selected cardiorespiratory variables onto PC_1_. Only the loading of FeO_2_ significantly decreased in Post-Et (*t* = 5.60; *p* = 0.001; *d* = 1.70) in participants who incremented the number of PCs, while no significant differences were found in loadings of participants who kept the number of PCs. PC_1_ was saturated by the loading of VE, FCO_2_, HR, SBP, and DBP, while PC_2_ was saturated by the projection of FeO_2_. Eigenvalues of PC_1_ significantly decreased in all participants from Test 1 to Test 2: *M* = 4.20, *SD* = 0.20 vs. *M* = 3.86, *SD* = 0.40, respectively (*t* = 2.95; *p* = 0.01; *d* = 1.08), even in those with no changes in the number of PCs. Accordingly, compared to Test 1, entropy was significantly higher in Test 2: *M* = 2.30, *SD* = 0.59 vs. *M* = 2.99, *SD* = 0.34 (*Z* = 2.33; *p* = 0.02; *d* = 1.43).

**Table 2:** Eigenvalues of PC1 and percentage of participants with one PC (PC_1_) and two PCs (PC_2_), in Test 1 and Test 2.

	**Test 1**			**Test 2**		
	**Eigenvalues (PC1)**	**% 1PC**	**% 2PCs**	**Eigenvalues (PC1)**	**% 1PC**	**% 2PCs**
Mean	4.20	60.00	40.00	3.86	0.00	100.00
*SD*	0.20	-	-	0.40	-	-

*% 1PC, percentage of participants with only one principal component (1PC); % 2PCs, percentage of participants with two principal components (2PCs)*.

**Table 3:** Means (standard desviations) of the projection of the selected cardiovascular and cardiorespiratory variables onto PC_1_.

	**Test 1**						**Test 2**					
	**VE**	**FeO_2_**	**FeCO_2_**	**HR**	**SBP**	**DBP**	**VE**	**FeO_2_**	**FeCO_2_**	**HR**	**SBP**	**DBP**
+N° PCs	0.94	0.71^*^	0.69	0.94	0.87	0.80	0.93	0.37^*^	0.73	0.88	0.86	0.75
	(0.01)	(0.15)	(0.18)	(0.02)	(0.06)	(0.10)	(0.02)	(0.24)	(0.09)	(0.09)	(0.05)	(0.14)
=N° PCs	0.93	0.41	0.74	0.94	0.91	0.85	0.93	0.37	0.81	0.93	0.92	0.82
	(0.03)	(0.29)	(0.32)	(0.04)	(0.06)	(0.07)	(0.01)	(0.18)	(0.11)	(0.07)	(0.05)	(0.10)

* *p < 0.05; +N° PCs, participants who increased the number of PCs in Test 2; =N° PCs, participants who kept the number of PCs in Test 2; VE, ventilation; FeO_2_, expired fraction of O2; FeCO_2_, expired fraction of CO_2_; HR, heart rate; SBP, systolic blood pressure; DBP, diastolic blood pressure*.

## Discussion

Based on the PCA approach and information entropy measures, a reduction of CRC was found in Test 2 compared to Test 1, without differences in maximal performance and cardiorespiratory variables in consecutive maximal exercises. The increased number of PCs, and/or the entropy during Test 2 means that the shared variability, i.e., co-variability, among the cardiorespiratory variables under study (VE, FeO_2_, FeCO_2_, HR, SBP, and DBP) decreased in Test 2. These effects on CRC should be carefully considered in cardiorespiratory exercise testing because they reflect a higher physiological strain and they have gone undetected through the commonly explored maximal performace (Wmax) and physiological variables (VEmax, VO_2_ max, HRmax, and VT).

As shown in Figure 1, the variance of the six studied cardio-respiratory variables was captured by a sole PC (PC_1_) in Test 1 (baseline conditions). However, a second PC emerged (PC_2_) in Test 2, i.e., five variables (VE, FeCO_2_, HR, SBP, and DBP) showed a large degree of co-variability (forming PC_1_) and only FeO_2_ was dominantly aligned with PC_2_. This means that Test 2 elicited a reduction of the correlation degree between FeO_2_ and the remaining variables. As previously described by Skinner and McLellan (1980), FeO_2_is reduced at exercise onset as a result of hyperventilation. The particular dynamics of FeO_2_ (decreasing at onset and increasing later) regarding the rest of variables forming PC_1_ (dominantly increasing during the test) was responsible for this lack of co-variation and the formation of PC_2_. In fact, the observed longer duration of FeO_2_decreasing phase in Test 2 was probably responsible for the formation of a new PC (PC_2_). This longer duration of the FeO_2_ decreasing phase could be explained by the large hyperventilation observed at the onset of Test 2 compared to Test 1 (baseline condition; see Table 1).

The results of the eigenvalues of PC_1_ and the information entropy measure (Haken, 2000), reinforced previous findings. PC_1_ eigenvalues, indicating the ratio of explained variances, were reduced in Test 2 and the information entropy was increased, showing a reorganization and diversification within the cardiorespiratory system. Entropy increased especially in those participants who incremented the numbers of PCs, since more states (i.e., more PCs) are available and more information was needed to specify the system's function.

In contrast with the current results, Balagué et al. (2016), found a reduction in the number of PCs during cardiorespiratory testing after a training period of 6 weeks (from two PCs before training to one PC after training) and pointed out to an improvement of CRC with training. Thus, while in the short-term a previous maximal exercise (Test 1) produced an increase in the number of PCs and a reduced CRC (Test 2), in the long-term (training effect) it seemed to have the opposite effect. These results are in agreement with the observed antagonist effect of workloads on performance in function of time. While in the short-term workloads produce a gain of strain and a corresponding impairment of performance, in the long-term they provoke workload tolerance and performance improvement (Perl, 2001).

While a reduction of CRC was revealed in Test 2 with respect to Test 1, no significant changes were found in maximal workload and physiological variables (VEmax, VO_2_ max, HRmax, and VT). The lack of differences in VO_2_ max was in agreement with previous results repeating graded and maximal cardiorespiratory tests with a 10 min recovery between tests (Hall-López et al., 2015). Due to the limited recovery time left to participants between tests in the current study (6 min), the initial values of cardiorespiratory variables were higher in Test 2 (see Table 1) confirming that participants performed the second test after an incomplete recovery. Although Test 2 started with altered initial conditions, this did not affect the maximal performance and physiological values attained, which did not differ from those registered in Test 1. In contrast, CRC was reduced in Test 2 compared to Test 1 showing that the cardiorespiratory system diversified and re-allocated its resources in order to satisfy the motor task demands. To this end, our results can be differently interpreted than those obtained by Hall-López et al. (2015) who stated that the cardiovascular demand had to be higher in the second test in order to reach the previous VO_2_ max. However, both studies are difficult to compare because they apply a different exercise, protocol and recovery time.

The lack of agreement among the commonly explored quantitative maximal performance and physiological variables and the qualitative results obtained through the evaluation of CRC in cardiorespiratory exercise testing has been previously reported (Balagué et al., 2016). Thus, the current findings give consistency to the claim of a higher sensitivity of CRC, not only in the evaluation of long-term effects of exercise (training effects), but also in the short-term effects tested here through consecutive maximal exercises. Future research should clarify however if the present results are a consequence of fatigue accumulation.

Six participants in this study had two PCs instead of one PC in the baseline conditions. Testing a similar type of population only formed by males, Balagué et al. (2016) found that most participants (80%) had two PCs instead of one PC (20%) in the baseline status. If the differences found in the number of PCs before intervention were due to gender or to a different fitness or health status of the participants should be further investigated. There is already some published evidence connecting aging and disease with alterations in CRC (Chang et al., 2013; García et al., 2013; Iatsenko et al., 2013). However, the concept of “cardiorespiratory coupling” used by these authors, which refers to the adjustment of heart beats at phases of the respiratory cycle (respiratory sinus arrhythmia; RSA), differs from the concept of CRC used in the current study. In fact, as indicated by Widjaja et al. (2015), RSA is in essence a measure of gain of the cardiorespiratory interaction and not of cardiorespiratory coupling; one could have differences in gain, but with a preserved coupling. The concept of CRC used in this research assumes a mutual influence of cardiovascular and respiratory oscillations leading to spontaneous coordination.

Regarding the methodological limitations of these results, it should be pointed out that PCA is a linear dimension reduction technique and, consequently, is only sensitive to linear correlational structure within the data. In future research it might be interesting, and even desirable, to apply other statistical techniques for dimension reduction used more generally in this respect, especially in relation to its nonlinear generalization, such as nonlinear PCA methods (Tenenbaum et al., 2000), or network component analysis (NCA; Liao et al., 2003).

From a practical point of view, it is recommended to evaluate CRC together with commonly registered maximal performance and cardiorespiratory variables to assess health and fitness status, and improve the interpretation of cardiorespiratory exercise testing. More specifically, given that changes in CRC seem more sensitive to a previous maximal exercise than gold standards, such as Wmax, VO_2_ max, or VT, they may contribute to prevention and early detection of exercise-induced fatigue effects, which are manifested at different time scales, including overreaching states or even overtraining syndrome, since it has been observed that their symptoms result from a bad coupling between physiological subsystems (Kreher, 2016) and are not easily recognized through common physiological tests (Meeusen et al., 2012). However, further research is warranted to clarify: 1/ the connection between the number of PCs and gender, health or training status, and 2/ the usefulness of CRC in cardiorespiratory exercise evaluation in different types of population.

In conclusion, the current study revealed a reduction in CRC after a consecutive repeated graded maximal cardiorespiratory test with no differences in the commonly explored maximal performance and physiological variables.

## Author contributions

SG, CJ, RH, JV, and NB conceived the paper and jointly drafted and reviewed the content; CJ worked on acquisition of the data; RH conceived the approach to data analysis. The authors approved the final version and agree to be accountable for all aspect of the work.

### Conflict of interest statement

The authors declare that the research was conducted in the absence of any commercial or financial relationships that could be construed as a potential conflict of interest.

## References

[B1] American Thoracic Society (2003). ATS/ACCP statement on cardiopulmonary exercise testing. Am. J. Respir. Crit. Care Med. 167, 211–277. 10.1164/rccm.167.2.211 12524257

[B2] Balagué N. González J. Javierre C. Hristovski R. Aragonés D. Álamo J.. (2016). Cardiorespiratory coordination after training and detraining. A principal component analysis approach. Front. Physiol. 7:35. 10.3389/fphys.2016.00035 26903884 PMC4751338

[B3] Balagué N. Hristovski R. García S. Aragonés D. Razon S. Tenenbaum G. (2014). Intentional thought dynamics during exercise performed until volitional exhaustion. J. Sports Sci. 33, 48–57. 10.1080/02640414.2014.921833 24870059

[B4] Bruce O. Moull K. Fischer S. (2017). Principal components analysis to characterise fatigue-related changes in technique: application to double under jump rope. J. Sports Sci. 35, 1300–1309. 10.1080/02640414.2016.1221523 27556961

[B5] Chang J. Kim E. Jung D. Jeong S. Kim Y. Roh M.. (2015). Altered cardiorespiratory coupling in young male adults with excessive online gaming. Biol. Psychol. 110, 159–166. 10.1016/j.biopsycho.2015.07.016 26253868

[B6] Chang J. Lee S. Ju G. Kim J. Ha K. Yoon I. (2013). Enhanced cardiorespiratory coupling in patients with obstructive sleep apnea following continuous positive airway pressure treatment. Sleep Med. 14, 1132–1138. 10.1016/j.sleep.2013.04.024 24051114

[B7] Denis D. J. (2016). Applied Univariate, Bivariate, And Multivariate Statistics. Hoboken, NJ: Wiley.

[B8] Eckert S. Hornskotte D. (2002). Comparison of Portapres non-invasive blood pressure measurement in the finger with intra-aortic pressure measurement during incremental bycicle exercise. Blood Press. Monit. 7, 179–183. 10.1097/00126097-200206000-00006 12131075

[B9] Faul F. Erdfelder E. Lang A. G. Buchner A. (2007). G^*^Power 3: a flexible statistical power analysis program for the social, behavioral, and biomedical sciences. Behav. Res. Methods 39, 175–191. 10.3758/BF03193146 17695343

[B10] García A. Jr. Koschnitzky J. E. Dashevskiy T. Ramirez J. M. (2013). Cardiorespiratory coupling in health and disease. Auton. Neurosci. 175, 26–37. 10.1016/j.autneu.2013.02.006 23497744 PMC3683976

[B11] Haken H. (2000). Information and Self-Organization. A Macroscopic Approach to Complex Systems. New York, NY: Springer.

[B12] Hall-López J. A. Ochoa-Martínez P. Y. Moncada-Jiménez J. Méndez M. A. O. García I. M. García M. A. M. (2015). Reliability of the maximal oxygen uptake following two consecutive trials by indirect calorimetry. Nutr. Hosp. 31, 1726–1732. 10.3305/nh.2015.31.4.8487 25795964

[B13] Hristovski R. Balagué N. (2010). Fatigue-induced spontaneous termination point-Nonequilibrium phase transitions and critical behavior in quasi-isometric exertion. Hum. Mov. Sci. 29, 483–493. 10.1016/j.humov.2010.05.004 20619908

[B14] Iatsenko D. Bernjak A. Stankovski T. Shiogai Y. Owen-Lynch P. J. Clarkson P. B. M.. (2013). Evolution of cardiorespiratory interactions with age. Philos. Trans. A Math. Phys. Eng. Sci. 371:20110622. 10.1098/rsta.2011.0622 23858485 PMC4042892

[B15] Jolliffe I. (2002). Principal Component Analysis. New York, NY: Springer.

[B16] Kreher J. (2016). Diagnosis and prevention of overtraining syndrome: an opinion on education strategies. Open Access J. Sports Med. 8, 115–122. 10.2147/OAJSM.S91657 PMC5019445 27660501

[B17] Latash M. L. (2008). Synergy. Oxford University Press.

[B18] Liao J. C. Boscolo R. Yang Y.-L. Tran L. M. Sabatti C. Roychowdhury V. P. (2003). Network component analysis: reconstruction of regulatory signals in biological systems. Proc. Natl. Acad. Sci. U.S.A. 100, 15522–15527. 10.1073/pnas.2136632100 14673099 PMC307600

[B19] Meeusen R. Duclos M. Foster C. Fry A. Gleeson M. Nieman D.. (2012). Prevention, diagnosis, and treatment of the overtraining syndrome: joint consensus statement of the European College of Sport Science and the American College of Sports Medicine. Med. Sci. Sports Exerc. 45, 186–205. 10.1249/MSS.0b013e318279a10a 23247672

[B20] Mizrahi J. Verbitsky O. Isakov E. Daily D. (2000). Effect of fatigue on leg kinematics and impact acceleration in long distance running. Hum. Mov. Sci. 19, 139–151. 10.1016/S0167-9457(00)00013-0

[B21] Naudts J. (2005). Boltzmann entropy and the microcanonical ensemble. Europhys. Lett. 69, 719–724. 10.1209/epl/i2004-10413-1

[B22] Perl J. (2001). PerPot: a metamodel for simulation of load performance interaction. Eur. J. Sport Sci. 1, 1–13. 10.1080/17461390100071202

[B23] Reinhard U. Müller P. H. Schmülling R. (1979). Determination of anaerobic threshold by the ventilation equivalent in normal individuals. Respiration 38, 36–42. 10.1159/000194056 493728

[B24] Scholz J. P. Schöner G. (1999). The uncontrolled manifold concept: identifying control variables for a functional task. Exp. Brain Res. 126, 289–306. 10.1007/s002210050738 10382616

[B25] Schulz S. Adochiei F. Edu I. Schroeder L. Costin H. Bär K.. (2013). Cardiovascular and cardiorespiratory coupling analyses: a review. Philos. Trans. A Math. Phys. Eng. Sci. 371:20120191. 10.1098/rsta.2012.0191 23858490

[B26] Seely A. J. E. Macklem P. (2012). Fractal variability: an emergent property of complex dissipative systems. Chaos 22, 19–22. 10.1063/1.3675622 22462984

[B27] Skinner J. S. McLellan T. M. (1980). The transition from aerobic to anaerobic metabolism. Res. Q. Exerc. Sport 1, 234–248. 10.1080/02701367.1980.10609285 7394286

[B28] Tenenbaum J. B. de Silva V. Langford J. C. (2000). A global geometric framework for nonlinear dimensionality reduction. Science 290, 1319–1323. 10.1126/science.290.5500.2319 11125149

[B29] Vázquez P. Hristovski R. Balagué N. (2016). The path to exhaustion: time-variability properties of coordinative variables during continuous exercise. Front. Physiol. 7:37. 10.3389/fphys.2016.00037 26913006 PMC4753307

[B30] Widjaja D. Montalto A. Vlemincx E. Marinazzo D. Van Huffel S. Faes L. (2015). Cardiorespiratory information dynamics during mental arithmetic and sustained attention. PLoS ONE 4:10 10.1371/journal.pone.0129112 PMC4456404 26042824

